# Determinants of perceived beneficial impact of a community-university partnership in an urban environment: a cross-sectional survey

**DOI:** 10.3389/fpubh.2025.1670334

**Published:** 2026-01-07

**Authors:** Kiparissenia Samara, Areti-Dimitra Koulouvari, Theodoros N. Sergentanis, Evanthia Sakellari, Constantina Skanavis, Areti Lagiou

**Affiliations:** Laboratory of Hygiene and Epidemiology, Department of Public and Community Health, School of Public Health, University of West Attica, Athens, Greece

**Keywords:** community engagement, community-university partnerships (CUP), CUP, Greece, health services

## Abstract

**Background:**

Community-University Partnerships (CUP) worldwide are a growing field in the collaboration between academia and local communities. In Greece, the University of West Attica, the second largest university in the metropolitan area of Athens, shapes pioneering CUP initiatives. The aim of the present study is to examine potential determinants of perceived beneficial impact of a CUP between the University and the local community in the urban setting.

**Methods:**

A cross-sectional study was conducted among 581 residents of the Egaleo municipality in the urban agglomeration of Athens, where the University is located (63.9% females). The perceived benefits of the CUP, on various life aspects of the community, were measured by a score derived from a previously validated self-administered 13-item questionnaire which recorded information on various potential determinants, such as sociodemographic and lifestyle variables, awareness and use of municipal and health services as well as evaluation of municipality services. Multivariate logistic regression analysis was conducted.

**Results:**

The median CUP benefits score was 35 (IQR: 30–39). Higher CUP scores, after adjustment for gender, age, and employment status, were significantly associated with higher educational attainment (adjusted OR = 1.59; 95% CI: 1.10–2.29; *p* = 0.013), engagement in recycling (adjusted OR = 2.28; 95% CI: 1.50–3.47; *p* < 0.001), and greater awareness about municipal services and facilities, such as gyms (adjusted OR = 1.73; 95% CI: 1.24–2.42; *p* = 0.001), the “Help at Home” program (adjusted OR = 1.60; 95% CI: 1.14–2.25; *p* = 0.007), and social services (adjusted OR = 1.47; 95% CI: 1.04–2.07; *p* = 0.028). Use of cultural centers also correlated with positive perceptions (adjusted OR = 1.72; 95% CI: 1.13–2.62; *p* = 0.012).

**Conclusion:**

Findings highlight the importance of education attainment, regular engagement in environmentally friendly practices—such as recycling—and awareness/utilization of municipal services and facilities in shaping positive community perceptions toward CUP initiatives. These results also emphasize the need for targeted outreach to less educated and disadvantaged groups within the community, to promote health equity, strengthen community involvement, and the long-term sustainability of CUP initiatives.

## Introduction

From community participation to community engagement and empowerment, decades of research in public health and health promotion have emphasized the impact of community involvement, ownership and action on the health and wellbeing of individuals and populations. Therefore, the development of strong partnerships between communities and various stakeholders, has gained great recognition as a valuable and effective working framework for advancing health, addressing health challenges and disparities, as well as tackling social inequalities (1–4).

Among others, research has highlighted the unique role of community-university partnerships (CUPs), indicating that reciprocal collaborations between academic institutions and local or broader communities can greatly benefit both parties (5–10). Universities are important partners in achieving aspects of community development and prosperity, fostering sustainable health improvements and equitable social change. Furthermore, research has shown that well-structured CUPs not only support applied research but also facilitate the dissemination of results that address actual health challenges. At the same time, CUPs promote the collaborative development of effective solutions and play a significant role in strengthening community capacity, enabling communities to improve their quality of life and respond more effectively to important public health challenges (5, 7–15).

Community-based participatory research (CBPR) has long served as a valuable framework to develop and apply CUPs; theory and practice on the subject has evolved, with a significant part of research focusing on the characteristics that enhance the effectiveness of partnerships, foster the sustainability and long-term impacts of the interventions and programs implemented (16–18). Within this process, exploring the views and perceptions of the community regarding the impact and other various aspects of CUPs is of great significance, as it can provide valuable practical recommendations regarding short- and long-term contributions and outcomes of CUPs (19).

Additionally, a key factor in the CUP process is the investigation of factors— including sociodemographic variables, lifestyle health-related perceptions and behaviors, as well as certain contextual and environmental factors, that may influence community members’ awareness, engagement, and willingness to participate in CUP initiatives (20–24). Certain pro-environmental behaviors, such as recycling, have been linked to broader patterns of civic participation, social responsibility, and community engagement. Furthermore, awareness and utilization of community and municipal services, which are commonly regarded as functional components of the broader concept of health literacy, offer a useful insight of how individuals access, interpret, and engage with health-related community resources. Integrating these factors into the study’s rationale supports the development of interventions that are not only embedded within community structures, but are also culturally appropriate and sensitive to the specific needs, social determinants, and characteristics of the targeted communities (25).

In Greece, CUP initiatives are currently a growing field. The University of West Attica (UNIWA) is the second largest university in the metropolitan area of Athens, the capital of Greece. UNIWA’s two largest campuses are located in the Municipality of Egaleo, in the urban agglomeration of Athens. A novel 13-item instrument has been developed and validated in order to measure the impact of CUP initiatives (26). The present study aims to examine the potential determinants of perceived benefits of a CUP between the University and the local community in the urban setting.

## Materials and methods

### Study design and questionnaire

This cross-sectional survey was conducted in Egaleo, West Attica, where the main campus of the University of West Attica (UNIWA) is located. The study was approved by the Ethics Committee of the University of West Attica (approval number: 70441/27-7-2022). The survey utilized a sample of 581 adults residents (18+) coming from respective randomly selected households. Data collection was conducted at participants’ homes, primarily in the afternoons. To ensure randomness and avoid duplicate responses, each interviewer was assigned to a specific neighborhood block and instructed to interview every 12th household. When multiple adults were present, the individual whose birthday (day and month) was closest to the interview date was selected.

Although the original sample was planned to include 582 individuals (1% of the adult population of 58,161), the final number of respondents reached was 644. However, 581 of the participants completed a full questionnaire (participation rate: 90.22%).

The survey questionnaire was administered in hard copy form; it was self-administered, but a well-trained interviewer was present in person as a bystander to resolve any difficulties or questions potentially stemming from insufficient literacy of the participants; notably, however, no problems pertaining to the comprehension of the questionnaire items were noted.

The validated self-administered CUP questionnaire recorded information on potential determinants, including sociodemographic characteristics, health related perceptions and behaviors, awareness and use of municipal and health services as well as evaluation of municipality services in order to assess the perception of participants of the benefits (impact and domain-specific contributions of the CUP) (26); higher scores denoted greater benefits perceived by the participants.

Regarding sociodemographic variables, the participants’ gender, education, marital status and professional status, were recorded. A cut-off of 12 years of education was selected to distinguish individuals who had completed secondary education from those with lower attainment, reflecting a meaningful difference in formal learning, access to information, and capacity for navigating community and health-related services. Lifestyle health-related perceptions and behaviors were assessed through items evaluating individuals’ views on personal health status, preventive health practices, and selected pro-environmental behaviors such as recycling. Information (yes or no) on awareness and use of several municipal infrastructures and services, including sports facilities, gyms, parks, city squares, children’s playgrounds, swimming pools, cultural center, community/open care centers for the older adults, daycare centers, “Ηelp at home” program, camping facilities, health and social services was also collected. Awareness and use of municipal facilities and services were used to assess whether general community engagement, familiarity with local health and social services, or broader patterns of civic participation influence positive perceptions of the University’s CUP activities, particularly those related to health and social wellbeing.

Furthermore, the evaluation of the municipality by the participants was recorded using a 4-level scale (very good; somehow good; not good; not at all good) regarding the municipal public spaces, sidewalk maintenance, public safety. The perceived changes during the previous three years were also evaluated using a 3-level scale (improved; stayed the same; deteriorated) regarding the quality of public recreational spaces, life in the Municipality overall, public transportation and public playgrounds.

### Statistical analysis

Descriptive statistics were estimated; categorical variables were summarized using frequencies and relative frequencies (%). Continuous variables were presented using the median and interquartile range (values of the first and third quartile). The perceived benefits of the CUP on the life aspects of the local community was calculated, as previously described in the validation study of the CUP questionnaire (26). The CUP questionnaire includes 13 items; formulated in a three-point Likert scale (1 representing “disagree,” 2 representing “neither agree nor disagree,” and 3 representing “agree”); the lowest possible value of the questionnaire is 13 and the highest possible is 39, the latter denoting a more pronounced, positive effect of the CUP (26). Two subscales/factors have been described, one pertaining to the overall impact of the CUP and one to the domain-specific contributions of the CUP (27); Cronbach’s alpha was calculated for the total score, as well as for each of the two subscales/factors.

Regarding inferential statistics, logistic regression analysis was undertaken with the CUP score treated as the dependent variable (> = median vs. <median); the CUP score was converted to a binary variable due to its marked deviation from normality (*p* < 0.00001, Kolmogorov–Smirnov test). At the univariate analysis, the recorded variables pertaining to sociodemographic status, awareness, use and evaluation of the Municipality services were set as independent variables, one by one. The categorization of independent variables in the analysis was *a priori* agreed upon by the study team, i.e., male vs. female for gender; ≥42 (median) vs. < 42 years for age; ≥12 vs. <12 years for education; married vs. single/divorced/widowed for marital status; in work or education vs. unemployed/household/retired for the professional status; ≥3 vs. <3 household members, self-owned car vs. bus/metro/any other regarding the way of going to work and overweight/obese vs. normal weight/underweight for the body weight status. The yes vs. no comparison was adopted for the remaining sociodemographic variables, as well as for all variables pertaining to the awareness and use of services. Regarding the variables pertaining to the evaluation of municipal services, the very good/somehow good vs. not good/not at all good comparison was uniformly adopted; for the variables evaluating differences in the municipal facilities during the last three years, the improved vs. deteriorated/stayed the same comparison was uniformly adopted.

All variables proven significant at the univariate analysis were entered into the multivariate logistic regression analysis, where estimates were adjusted for gender, age, education and professional status.

The results of the logistic regression analyses were presented as odds ratios (OR) with their 95% confidence intervals (95% CI). The level of statistical significance was set at 0.05. The statistical analysis was conducted with STATA version 16 (Stata Corp, College Station, TX, United States).

## Results

### Description of the study sample

The description of the study sample is shown in Table 1. A total of 581 participants were included in the study; the majority were females (63.9%, *n* = 371). In terms of educational attainment, 27.9% (*n* = 162) of participants reported only secondary/high school education, 18.6% (*n* = 108) held a university degree, 18.2% (*n* = 106) had graduated from a technical university, and 17.6% (*n* = 102) from a post-secondary technical school. A smaller proportion held postgraduate degrees (10.7% having an MSc and 1.2% a PhD).

**Table 1:** Description of the categorical variables in the study sample (*n* = 581).

Sociodemographic and lifestyle variables	n (%)
Gender	
Male	210 (36.1)
Female	371 (63.9)
Education	
Never gone to school / Partly primary school (<6 years)	8 (1.4)
Primary school (6 years)	26 (4.5)
Secondary/high school (6–12 years)	162 (27.9)
Post-secondary technical school	102 (17.6)
Technical university	106 (18.2)
University	108 (18.6)
MSc degree	62 (10.7)
PhD degree	7 (1.2)
Marital status	
Married, with children	306 (52.7)
Married, without children	38 (6.5)
Single living alone or in a partnership	88 (15.2)
Single living with parents	57 (9.8)
Single living with other relatives	17 (2.9)
Divorced	51 (8.8)
Widowed	24 (4.1)
Professional status	
Self-employed	65 (11.2)
Employer	24 (4.1)
Salaried, full-time	334 (57.5)
Salaried, part-time	49 (8.4)
Unemployed	35 (6.0)
Household	22 (3.8)
Student	14 (2.4)
Retired	38 (6.5)
Way of going to work	
Self-owned car	293 (50.4)
City bus	60 (10.3)
Intercity bus	13 (2.2)
Metro	108 (18.6)
Other	107 (18.4)
Body weight status	
Underweight	11 (1.9)
Normal weight	244 (42.0)
Overweight	253 (43.6)
Obese	73 (12.6)
Current smoking	
Yes, every day	186 (32.0)
Yes, occasionally	47 (8.1)
No	348 (59.9)
Setting where health care is received	
Private health care / private doctor offices, exclusively or in conjunction with public health care	457 (78.7)
Public health care only	124 (21.3)
Private insurance	
Yes	120 (20.6)
No	461 (79.4)
Disability	
Yes	6 (1.0)
No	575 (99.0)
Disease during the previous 12 months	
Yes	147 (25.3)
No	434 (74.7)
Use of chronic medication	
No	358 (61.6)
One agent	109 (18.8)
2–3 agents	85 (14.6)
4–5 agents	19 (3.3)
5 + agents	10 (1.7)
Participation in health promotion programs during the recent 5 years	
Yes	79 (13.6)
No	502 (86.4)
Engagement in recycling	
Yes	458 (78.8)
No	123 (21.2)
Variables pertaining to the awareness of participants	
Awareness about the availability of	
Sports facilities of the Municipality	507 (87.3)
Gyms of the Municipality	313 (53.9)
Parks of the Municipality	550 (94.7)
City squares of the Municipality	565 (97.3)
Children’s playgrounds of the Municipality	542 (93.3)
Swimming pools of the Municipality	498 (85.7)
Cultural center of the Municipality	474 (81.6)
Community/Open Care Centers for the Older adults of the Municipality	514 (88.5)
Daycare Centers of the Municipality	544 (93.6)
“Help at Home” Program of the Municipality	261 (44.9)
Camping of the Municipality	121 (20.8)
Health Services of the Municipality	311 (53.5)
Social Services of the Municipality	350 (60.2)
Variables pertaining to the use of municipal facilities by the participants	
Use of	
Sports facilities of the Municipality	209 (36.0)
Gyms of the Municipality	55 (9.5)
Parks of the Municipality	370 (63.7)
City squares of the Municipality	333 (57.3)
Children’s playgrounds of the Municipality	190 (32.7)
Swimming pools of the Municipality	103 (17.7)
Cultural center of the Municipality	122 (21.0)
Community/Open Care Centers for the Older adults of the Municipality	61 (10.5)
Daycare Centers of the Municipality	151 (26.0)
“Help at Home” Program of the Municipality	16 (2.7)
Camping of the Municipality	11 (1.9)
Health Services of the Municipality	126 (21.7)
Social Services of the Municipality	116 (20.0)
Evaluation of the municipality by the participants	
Evaluation of municipal public spaces in the neighborhood	
Very good	49 (8.4)
Somehow good	226 (38.9)
Not good	195 (33.6)
Not at all good	111 (19.1)
Evaluation of sidewalk maintenance in the neighborhood	
Very good	19 (3.3)
Somehow good	142 (24.4)
Not good	229 (39.4)
Not at all good	191 (32.9)
Evaluation of public safety in the neighborhood	
Very good	15 (2.6)
Somehow good	141 (24.3)
Not good	290 (49.9)
Not at all good	135 (23.2)
During the last three years, the quality of public recreational spaces has	
Improved	88 (15.2)
Deteriorated	158 (27.2)
Stayed the same	335 (57.7)
During the last three years, the quality of life in the Municipality (regarding unemployment, safety, housing, natural environment) has	
Improved	58 (10.0)
Deteriorated	197 (33.9)
Stayed the same	326 (56.1)
During the last three years, the quality of public transportation in the Municipality has	
Improved	93 (16.0)
Deteriorated	124 (21.3)
Stayed the same	364 (62.7)
During the last three years, the quality of public playgrounds and spaces for children has	
Improved	82 (14.1)
Deteriorated	213 (36.7)
Stayed the same	286 (49.2)

Regarding marital status, most participants were married with children (52.7%, *n* = 306). The majority of participants were salaried full-time employees (57.5%, *n* = 334), followed by self-employed individuals (11.2%, *n* = 65) and part-time salaried workers (8.4%, *n* = 49), whereas 6.5% (*n* = 38) had retired and 6.0% (*n* = 35) were unemployed. Concerning commuting, half of the participants (50.4%, *n* = 293) reported using a self-owned car to go to work; public transportation modes included the metro (18.6%, *n* = 108), city bus (10.3%, *n* = 60), and intercity bus (2.2%, *n* = 13), while 18.4% (*n* = 107) reported using other means.

In terms of body weight status, 43.6% of the participants were overweight (*n* = 253), 42.0% had normal weight (*n* = 244), 12.6% were obese (*n* = 73), and 1.9% (*n* = 11) were underweight. Smoking was fairly common among participants, with 32.0% (*n* = 186) reporting daily smoking and 8.1% (*n* = 47) smoking occasionally.

Most participants (78.7%, *n* = 457) used private health care services (exclusively or in conjunction with public services), while 21.3% (*n* = 124) relied solely on public health care. 20.6% (*n* = 120) were insured privately. Disability was reported by 1.0% of the sample; about a quarter (25.3%, *n* = 147) reported experiencing a disease during the previous 12 months. The use of chronic medication was also common: 18.8% (*n* = 109) used one agent, 14.6% (*n* = 85) used 2–3 agents, and 5.0% used four or more agents.

A large proportion (78.8%, *n* = 458) reported engaging in recycling. Only 13.6% of the sample had participated in health promotion programs in the past five years.

Participants showed high levels of awareness about the municipal infrastructures, particularly for city squares (97.3%, *n* = 565), parks (94.7%, *n* = 550), and children’s playgrounds (93.3%, *n* = 542), sports facilities (87.3%, *n* = 507). Awareness was lower for the “Help at Home” program (44.9%, *n* = 261) and municipal camping services (20.8%, *n* = 121).

Actual use of these facilities was more limited. The most frequently used infrastructures were parks (63.7%, *n* = 370) and city squares (57.3%, *n* = 333). Sports facilities were used by 36.0% (*n* = 209) and playgrounds by 32.7% (*n* = 190). Use was least frequent for the “Help at Home” program (2.7%) and municipal camping (1.9%).

Evaluation of municipal services showed that only 8.4% (*n* = 49) of respondents rated public spaces in their neighborhood as “very good,” while 38.9% (*n* = 226) found them “somehow good.” Regarding sidewalk maintenance, the majority reported a rather negative perception, with 39.4% rating that as “not good” (*n* = 229) and 32.9% (*n* = 191) as “not at all good.” Similarly, public safety received low evaluations, with 49.9% (*n* = 290) rating it as “not good” and 23.2% (*n* = 135) as “not at all good.”

Perceptions changes over the past three years varied. Most participants reported rather same conditions in public recreational spaces (57.7%, *n* = 335), quality of life (56.1%, *n* = 326), public transportation (62.7%, *n* = 364), and children’s spaces (49.2%, *n* = 286). However, 27.2% (*n* = 158) reported that the quality of public recreational spaces had deteriorated, approximately one third (33.9%, *n* = 197) reported an overall deterioration in the quality of live in the municipality, 36.7% (*n* = 213) believed that playgrounds and children’s spaces had deteriorated, and 21.3% (*n* = 124) believed the quality of public transportation in the municipality had worsened.

**Figure 1:**
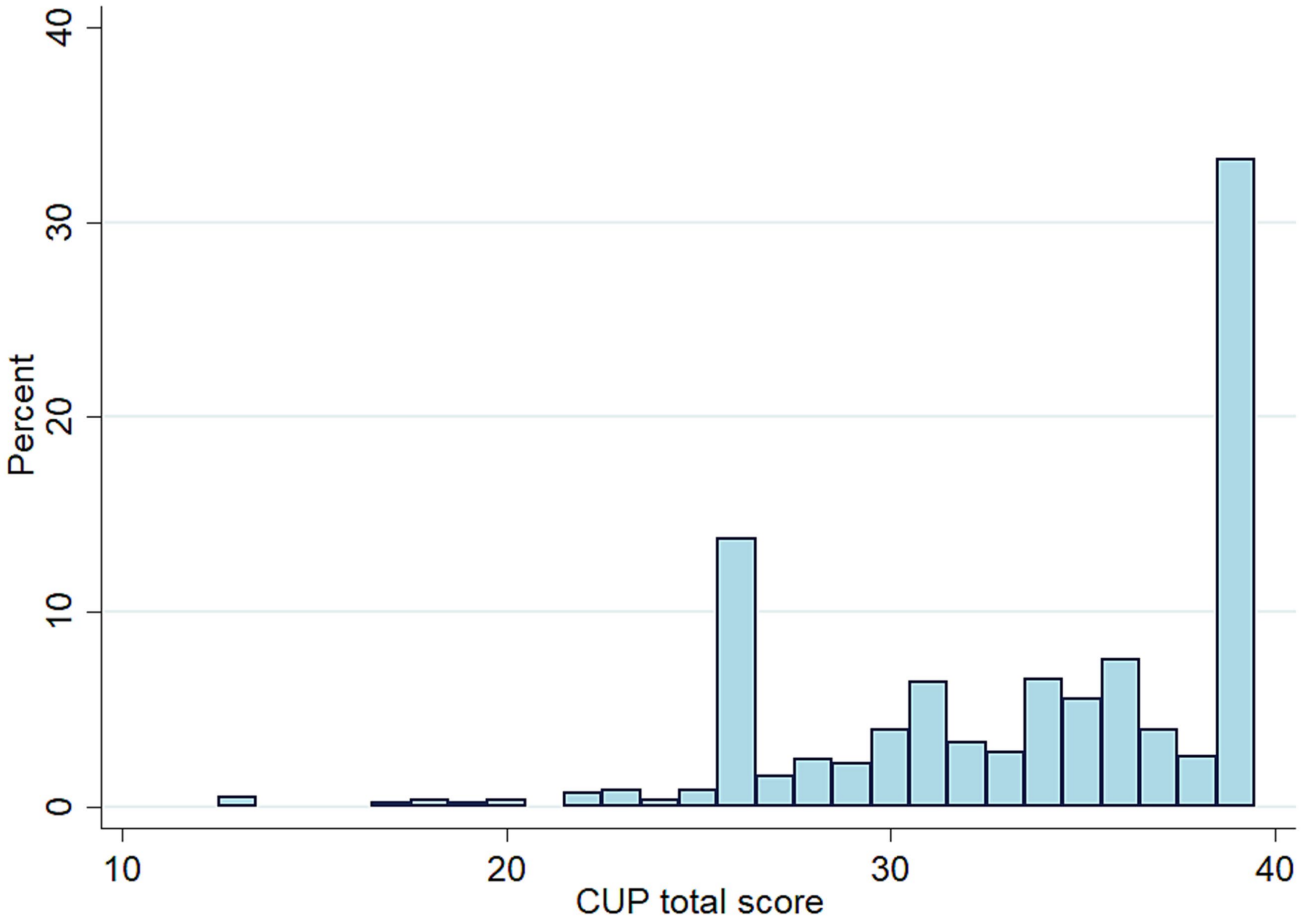
Chart presenting the CUP total score distribution in the study sample.

Table 2 summarizes the continuous variables of the study. The median age was 42 years (IQR: 34 to 54 years) and the median number of individuals living in the same household was 3 (IQR: 2 to 4 people). The median of the CUP total score was 35 (IQR 30 to 39), whereas the median value for the subscale 1 (overall impact of the CUP) was 14 (IQR: 11 to 15) and for the subscale 2 (domain-specific contributions of the CUP) was 21 (IQR: 17 to 24). Cronbach’s alpha indicated high reliability (alpha = 0.940 for the total score; 0.908 for subscale 1 and 0.916 for subscale 2). The distribution of the CUP total score in the study sample is presented in Figure 1.

**Table 2:** Description of the numeric variables in the study sample (*n* = 581).

Sociodemographic numeric variables	Median (IQR)
Age (years)	42 (34–54)
Members living in household	3 (2–4)
Questionnaire assessing CUP effects	
CUP total score	35 (30–39)
Subscale 1 (overall impact)	14 (11–15)
Subscale 2 (domain-specific contributions)	21 (17–24)

IQR, interquartile range.

### Results of the logistic regression analysis

Table 3 presents the results of the multivariate logistic regression analysis examining factors associated with participants’ perceived beneficial impact of the CUP initiative. After adjusting for gender, age, and professional status in the multivariate model, several variables remained independently associated with higher odds of reporting a beneficial impact of the CUP.

**Table 3:** Logistic regression analysis evaluating independent associations of the study variables with the CUP score (≥median vs. <median).

Variables	Category or increment	Univariate OR (95% CI)	*p*-value	Multivariate OR (95% CI)§	*p*-value
Sociodemographic and lifestyle variables					
Gender	Male vs. female	1.20 (0.85–1.68)	0.297		
Age	≥42 vs. < 42 years	0.97 (0.70–1.34)	0.832		
Education	≥12 vs. <12 years	**1.72 (1.22–2.44)**	**0.002**	**1.59 (1.10–2.29)**	**0.013**
Marital status	Married vs. single / divorced / widowed	1.17 (0.89–1.54)	0.257		
Professional status	In work or education vs. unemployed/household/retired	**1.77 (1.16–2.71)**	**0.008**	1.51 (0.97–2.35)	0.065
Household members	≥3 vs. <3	1.27 (0.90–1.78)	0.173		
Way of going to work	Self-owned car vs. bus/metro/any other	**1.44 (1.04–2.00)**	**0.029**	1.28 (0.91–1.81)	0.152
Body weight status	Overweight/obese vs. normal weight/underweight	1.02 (0.74–1.42)	0.901		
Current smoking	Yes vs. no	0.94 (0.67–1.31)	0.720		
Private health care	Yes vs. no	1.31 (0.88–1.95)	0.187		
Disability	Yes vs. no	0.89 (0.18–4.45)	0.889		
Disease during the previous 12 months	Yes vs. no	1.01 (0.70–1.47)	0.950		
Private insurance	Yes vs. no	**1.51 (1.00–2.27)**	**0.050**	1.35 (0.88–2.05)	0.165
Use of chronic medication	Yes vs. no	1.23 (0.88–1.73)	0.221		
Participation in health promotion programs during the recent 5 years	Yes vs. no	1.29 (0.80–2.08)	0.303		
Engagement in recycling	Yes vs. no	**2.42 (1.60–3.66)**	**<0.001**	**2.28 (1.50–3.47)**	**<0.001**
Awareness about availability of municipal facilities					
Sports facilities	Yes vs. no	1.56 (0.95–2.54)	0.078		
Municipal gyms	Yes vs. no	**1.68 (1.21–2.34)**	**0.002**	**1.73 (1.24–2.42)**	**0.001**
Parks	Yes vs. no	1.05 (0.51–2.17)	0.888		
City squares	Yes vs. no	1.12 (0.42–3.04)	0.818		
Children’s playgrounds	Yes vs. no	0.68 (0.35–1.33)	0.262		
Municipal swimming pools	Yes vs. no	1.24 (0.78–1.98)	0.360		
Municipal cultural center	Yes vs. no	1.41 (0.93–2.15)	0.107		
Community/Open Care Centers for the Older adults	Yes vs. no	1.18 (0.71–1.96)	0.532		
Municipal Daycare Centers	Yes vs. no	1.20 (0.61–2.33)	0.598		
The “Help at Home” Program	Yes vs. no	**1.48 (1.07–2.06)**	**0.019**	**1.60 (1.14–2.25)**	**0.007**
Municipal Camping	Yes vs. no	1.05 (0.70–1.56)	0.828		
Municipal Health Services	Yes vs. no	1.38 (0.99–1.92)	0.052		
Municipal Social Services	Yes vs. no	**1.42 (1.01–1.98)**	**0.041**	**1.47 (1.04–2.07)**	**0.028**
Use of municipal facilities					
Sports facilities	Yes vs. no	1.11 (0.79–1.56)	0.537		
Municipal gyms	Yes vs. no	1.63 (0.92–2.91)	0.094		
Parks	Yes vs. no	1.29 (0.92–1.81)	0.143		
City squares	Yes vs. no	1.12 (0.81–1.56)	0.497		
Children’s playgrounds	Yes vs. no	0.93 (0.66–1.31)	0.671		
Municipal swimming pools	Yes vs. no	**1.59 (1.03–2.46)**	**0.038**	1.51 (0.97–2.35)	0.071
Municipal cultural center	Yes vs. no	**1.78 (1.18–2.69)**	**0.006**	**1.72 (1.13–2.62)**	**0.012**
Community/Open Care Centers for the Older adults	Yes vs. no	**0.54 (0.32–0.94)**	**0.027**	0.75 (0.41–1.35)	0.333
Municipal Daycare Centers	Yes vs. no	1.12 (0.77–1.63)	0.543		
The “Help at Home” Program	Yes vs. no	0.69 (0.25–1.87)	0.463		
Municipal Camping	Yes vs. no	0.50 (0.15–1.74)	0.278		
Municipal Health Services	Yes vs. no	0.83 (0.56–1.23)	0.356		
Municipal Social Services	Yes vs. no	0.76 (0.51–1.15)	0.191		
Evaluation of the municipality infrastructures					
Municipal public spaces in the neighborhood	Very good/somehow good vs. not good/not at all good	1.11 (0.80–1.54)	0.539		
Sidewalk maintenance in the neighborhood	Very good/somehow good vs. not good/not at all good	0.78 (0.55–1.13)	0.190		
Public safety in the neighborhood	Very good/somehow good vs. not good/not at all good	0.72 (0.50–1.04)	0.078		
Changes during the last three years in the quality of					
Public recreational spaces	Improved vs. Deteriorated/stayed the same	0.97 (0.78–1.21)	0.778		
Life in the Municipality	Improved vs. Deteriorated/stayed the same	0.90 (0.71–1.15)	0.412		
Public transportation in the Municipality	Improved vs. Deteriorated/stayed the same	0.90 (0.73–1.12)	0.367		
Public playgrounds and spaces for children	Improved vs. Deteriorated/stayed the same	0.99 (0.79–1.24)	0.926		

Bold cells denote associations with *p* < 0.05. §adjusted for gender, age, education and professional status.

**Sociodemographic and lifestyle variables**: Educational attainment emerged as a significant independent determinant; participants with educational attainment of 12 or more years had higher odds of perceiving the CUP as beneficial (adjusted OR = 1.59; 95% CI: 1.10–2.29; *p* = 0.013). Moreover, individuals engaging in regular recycling had more than twice the odds of perceiving the CUP as beneficial (adjusted OR = 2.28; 95% CI: 1.50–3.47; *p* < 0.001).

Although the univariate analysis showed that professional status (univariate OR = 1.77; 95% CI: 1.16–2.71), going to work with a self-owned car (univariate OR = 1.44; 95% CI: 1.04–2.00) and private insurance (univariate OR = 1.51; 95% CI: 1.00–2.27) were associated with perception of CUP as beneficial, at the multivariate analysis, those associations lost significance; regarding professional status, only a marginal trend remained (*p* = 0.065).

**Awareness and use of Municipal facilities**: Several indicators of community engagement and familiarity with local services demonstrated higher odds of perceiving the CUP as beneficial. Awareness of municipal facilities and services including municipal gyms (adjusted OR = 1.73; 95% CI: 1.24–2.42; *p* = 0.001), the “Help at Home” program (adjusted OR = 1.60; 95% CI: 1.14–2.25; *p* = 0.007), and social services (adjusted OR = 1.47; 95% CI: 1.04–2.07; *p* = 0.028) were significantly associated with positive perceptions of the CUP.

The use of the municipal cultural center was independently associated with positive CUP perception (adjusted OR = 1.72; 95% CI: 1.13–2.62; *p* = 0.012). On the other hand, the use of municipal swimming pools exhibited only a borderline trend (*p* = 0.071) at the multivariate analysis. An inverse association with community/open care centers for the older adults (*p* = 0.027, univariate analysis) dissipated at the multivariate analysis (*p* = 0.333).

**Evaluation of municipality infrastructures and changes during the previous three years**: CUP perception was not associated with the evaluation of municipal public spaces, sidewalk maintenance, public safety in the neighborhood, or changes during the previous three years in the quality of public recreational spaces, life in the Municipality, transportation and public playgrounds for children.

## Discussion

This study highlighted that participants with higher levels of educational attainment, stronger involvement in environmentally friendly behaviors, such as recycling, and higher levels of awareness/use of available municipal facilities and services, had significantly more positive attitudes toward the CUP program, scoring higher on the CUP scale.

On the other hand, sociodemographic variables, such as gender, age, marital status, body weight, chronic medication use, disability, were not significantly associated with CUP scores, highlighting the importance of the aforementioned factors over individual demographic or clinical characteristics.

Educational attainment was identified as a key factor, associated with positive perceptions about the CUP in both univariate and multivariate analyses. In agreement with our finding, a higher level of education has consistently been associated not only with improved health outcomes (27, 28) but also with more informed, engaged, and proactive citizens within their community (29). Previous studies have supported this notion, showing that individuals with higher education tend to be more informed about available resources and services in their community and more motivated to participate in collaborative civic initiatives, such as CUPs (16, 30, 31).

In addition, an association between anticipated CUP benefits and pro-environmental behaviors such as recycling, was evident in our sample. Participants who recycled were over twice as likely to report favorable perceptions of the CUP compared to those who did not engage in recycling. Even after adjusting for certain demographic factors, recycling remained a strong independent predictor. Recycling behavior, beyond reflecting environmental responsibility, may also serve as a proxy for broader civic engagement, social responsibility, and pro-social norms, which could predispose individuals to view collaborative community initiatives, such as CUP activities, more positively. This is in agreement with previous studies having shown that certain friendly environmental behaviors, including recycling, often correlate with stronger involvement in community initiatives and a better sense of social cohesion (32–34).

The study also revealed that participants with both greater awareness and use of the available municipal services, reported more favorable views of the CUP. Positive perceptions about the CUP can be inscribed into a health literacy-related context. Although health literacy was not measured with a dedicated instrument in this survey, these variables can be viewed as functional proxies, reflecting residents’ capacity to access, understand, and engage with local health and social resource in a way that promotes health and wellbeing (35–37). It has been well documented that health literacy positively affects community participation, informed decision making, health services utilization, and overall wellbeing and empowerment (38).

All the above findings also highlight key issues that should be taken into consideration when aiming for a greater engagement of local communities into CUPs. Since higher educational attainment was a factor significantly associated with positive CUP perceptions, evidently the less educated individuals were less favorable toward the CUP. Strategies aimed at raising awareness about CUP should therefore focus on the less privileged individuals, such as those with lower educational attainment. Hence, it seems of great importance to further enhance outreach strategies for those members of the community, as they not only tend to be less motivated to participate in CUP initiatives but are also overall more vulnerable to health disparities and underutilization of community services (39–41).

To address those challenges, organizing targeted awareness campaigns focused on informing less privileged, vulnerable individuals about the value and benefits of CUPs could help improve their perceptions and encourage community involvement. Research findings have shown the positive impact of CUPs in enhancing health literacy across different community settings, providing strong evidence to advocate for such initiatives (42–44). In terms of building health literacy, the role of CUPs can be crucial; CUPs should be based on the real-life needs of the community, taking into account social determinants such as age, sex, ethnicity, socioeconomic status, housing, income and education level (45–48). In the scope of CUPs, health literacy should be viewed as an ongoing process of mutual and respective interaction, communication and collaboration with the communities; factors that reinforce health literacy and barriers that may hinder its development should also be considered (49).

The study has certain strengths, including a large random sample of the urban population of the metropolitan area of Athens, Greece, and the application of the previously validated tool designed to effectively evaluate the impact of the CUP within the local community (26). Additionally, the current study has highlighted significant associations with key determinants of positive perceptions about the CUP.

Despite the strengths, there are also certain limitations. Most importantly, the cross-sectional design of the study limits its ability to establish causal relationships, whereas the fact that the findings are based on a community sample in Egaleo may not reflect the view and perceptions of communities in different geographic locations or cultural contexts, and hence limiting the generalizability of the results to other regions or countries. Furthermore, no weighting of variance with respect to local demographics, or evaluation of potential clustering (due to assignment of interviewers to specific neighborhood blocks) has been undertaken. In addition, the conversion of the CUP score to a binary dependent variable might have led to the loss of information; notably however, significant associations still arose in the logistic regression analysis. Moreover, this survey was based on self-report questionnaire data; social desirability bias cannot be precluded in the responses of participants.

## Conclusion

This study has highlighted several key determinants of the perceived impact and benefits of the CUP in the Egaleo municipality. Higher educational attainment, increased participation and engagement in environmental behaviors such as recycling, and greater awareness and use of various municipal services were identified as the strongest correlates of favorable views toward the CUP. These results emphasize the need for focused outreach and awareness initiatives to enhance acceptance and participation in CUP programs, especially among the disadvantaged or less educated groups. By identifying the factors that shape public views, academia and local communities can better collaborate into building partnerships that are more inclusive and beneficial for everyone in the community.

## Data Availability

The raw data supporting the conclusions of this article will be made available by the authors, without undue reservation.
